# The Role of the Sedimentary Regime in Shaping the Distribution of Subtidal Sandbank Environments and the Associated Meiofaunal Nematode Communities: An Example from the Southern North Sea

**DOI:** 10.1371/journal.pone.0109445

**Published:** 2014-10-08

**Authors:** Michaela Schratzberger, Piers Larcombe

**Affiliations:** 1 Centre for Environment, Fisheries and Aquaculture Science, Lowestoft Laboratory, Lowestoft, Suffolk, United Kingdom; 2 RPS MetOcean, Subiaco, Western Australia, Australia; Scottish Association for Marine Science, United Kingdom

## Abstract

We combined sediment and faunal data to explore the role of the sedimentary regime in shaping the distribution of subtidal sandbank environments and the associated meiofaunal nematode communities at Broken Bank and Swarte Bank, in the southern North Sea. A variety of sediment transport processes occur in the area, differing in the frequency and magnitude of sediment mobility, and the continuum between erosion, translation and sediment accumulation. The seabed contained a variety of bedforms, including longitudinal furrows, and small to very large sandwaves. The bed sediments were dominated by fine and medium sands, with admixtures of silt and gravel. Based on sedimentary bedforms and grain size analysis, a total of 11 sedimentary facies were delineated, of which 8 were analysed in detail for their relationships with the meiofauna. The sedimentary facies fell clearly into groups of facies, respectively representing high, high-moderate and moderate, and episodic sediment mobility. For those sedimentary facies where daily movement of sediments and bedforms occurred (‘high’ sediment mobility), the resulting spatially homogeneous environments were dominated by an impoverished nematode community comprising small deposit feeders and large predators. Resistance to sediment movement and the ability to exploit alternative food sources were prominent functional features of the successful colonisers. Those facies characterised by relatively infrequent sediment mobility (‘episodic’ and ‘high-moderate and moderate’ sediment mobility) comprised a heterogeneous suite of benthic habitats, containing taxonomically and functionally diverse assemblages of nematodes of various sizes, feeding types and reproductive potential. Faunal distribution patterns here indicated trade-offs between the resistance to sediment movement, environmental tolerance and competitive abilities. Our focus on diverse assemblages of organisms with high turnover times, inhabiting highly dynamic sedimentary environments, has revealed new animal-sediment relationships of relevance to pure and applied science.

## Introduction

Scientists, socio-economists, environmental managers, policy-makers and the public all have interests in marine environmental studies and their outcomes. A common motivation of marine studies is the implicit or explicit search for indicators of natural and anthropogenic change, with an aim of identifying and assessing human impacts and the effects of management actions. As an example policy driver, the EU Habitats Directive for the protection of species and habitats [1] requires that management plans be created in order to detect and respond to human-induced change. In particular, such plans are to be prepared for the candidate Special Areas of Conservation (SAC), including subtidal mobile sandbanks, on which we expand below. Thus, it is a requirement that plans are able to detect human-induced change as distinct from the various types and timescales of natural variability, and that actions are identified which are able to be enacted to counter such (assumed) detrimental change. In most environments this is not a simple task, but in the dynamic marine sedimentary environment of much of the western European shelf it appears to be a major challenge indeed.

Effective conservation of marine habitats in European seas requires specific conservation objectives. These need to be achieved by closely linked management measures that regulate the activities of those marine industries which may threaten the integrity of the habitat, or the viability of the associated populations [2]. Conservation objectives for subtidal sandbanks generally demand that, subject to natural processes, the extent and distribution of sandbank habitats, their faunal structure and function, their gross morphology (e.g. depth, distribution and profile) and the supporting processes on which sandbank habitats rely, are maintained or recovered [3]. In order to be able to measure the success or otherwise of such management goals, detailed investigations of the links between the sedimentary habitat and the biota are essential.

Reviewing the literature on animal-sediment relationships, [4] found little evidence that animal distributions are determined by any of the sediment variables derived from grain size alone. Indeed, [5] summarised the results of nine recent studies, which used abiotic variables such as percentage mud, percentage calcium carbonate, percentage gravel, total organic carbon content, a bed disturbance factor (named seabed exposure by [6]), water depth, latitude, longitude, slope, turbidity and distance to ocean. In these studies, between 25 and 75% of variability in faunal distribution patterns was accounted for; most of the stronger relationships involved using physical measures of the sea floor and sediments. Hence, meaningful and predictive explanations for faunal distributions are likely to emerge if these are evaluated relative to the suite of hydrodynamic and sediment transport processes that are responsible for sediment distributions. It is also important to understand the physical form of the sediments (bathymetry and the nature of sedimentary bedforms) at a range of spatio-temporal scales, and the details of the grain size distribution.

A general appreciation of the highly heterogeneous nature of continental shelf sediments has progressively emerged in recent decades. Multi-beam bathymetric surveys have revealed numerous types of sedimentary and geomorphological features on the sea floor that occur at a variety of spatial scales, ranging from a few meters to several kilometres [7], [8], [9], [10]. Ground-truthing acoustic data to confirm the nature of the seabed is an important process, but the next step of improving understanding of the relationships with the fauna is hampered by gaps in our knowledge. These gaps include the difficulty of identifying organisms to fine taxonomic levels [11] and the comparative shortage of information on those organisms with a range of life-history characteristics which tie them closely to the sediment in which they live, such as meiofauna (comprising invertebrates that are retained on a 500–1000 µm mesh; [12]).

There are obvious possible dependencies of meiofauna upon the distribution and mobility of sediments [13], [14], [15]. When patches of suitable habitat are spatially or temporally discontinuous, an organism's dispersal capability will greatly affect its ability to colonise available space. Where the dispersal mechanism is closely related to the transport mechanisms of the associated sediment (e.g. meiofaunal nematodes) we would expect that the distribution and location of the sediment to be a key factor in controlling faunal distribution patterns. Mobile sediments at subtidal sandbanks could affect meiofaunal assemblages by altering the composition of sedimentary environments, thereby changing their suitability for settling organisms and/or by increasing the resuspension of organisms as a result of sediment deposition and/or sediment instability.

A detailed description of the benthic fauna associated with two linear sandbanks off the North Norfolk coast in the southern North Sea by [16] provided a general overview of the benthic ecology of the area. Here, we further explore these data, taking account of the topographic complexity of the surveyed sandbank habitats, to improve our understanding of the dynamics of the sandbank ecosystem. As such, this scoping study has two objectives: 1) to qualitatively describe the nature and origins of several kinds of sedimentological features and processes at two subtidal sandbanks in the southern North Sea, explicitly taking account of hydrodynamics and sediment transport processes, the bathymetry and nature of sedimentary bedforms, and grain size distributions and 2) to depict the taxonomic and functional characteristics of meiofaunal nematode communities which occupy these environments. We use our findings to better understand the way in which the physical dynamics of the seabed and its spatial heterogeneity might combine to determine the number and type of available niches and hence influence the diversity and composition of meiofaunal nematode communities.

## Field Surveys and Sample Processing

All data reported in this article were collected by the Centre for Environment, Fisheries and Aquaculture Science (Cefas), as part of a government-funded research project. As an executive agency of the UK Government, Cefas has an exemption from The Crown Estate (i.e. land owner of our study site) to collect sediment and other scientific material (except vertebrates) in UK waters. The activities involved in the survey of the seabed and the collection of benthic invertebrates were not subject to specific regulation and did not involve endangered or protected species. Throughout the survey, scientists and ship crew acted in a responsible and environmentally friendly manner.

We analysed the bathymetry, sedimentary bedforms, sediments and meiofaunal nematodes at and near Broken Bank (BB) and Swarte Bank (SB), in the southern North Sea, to examine the association of faunal assemblages and their sedimentary habitat. The sampling strategy was not designed to fully characterise nematode-sediment relationships at the two sandbanks, but rather to enable us, with minimal cost and effort, to make comparisons between nematode communities inhabiting a diverse range of sedimentary environments.

### Study site

**Figure 1:**
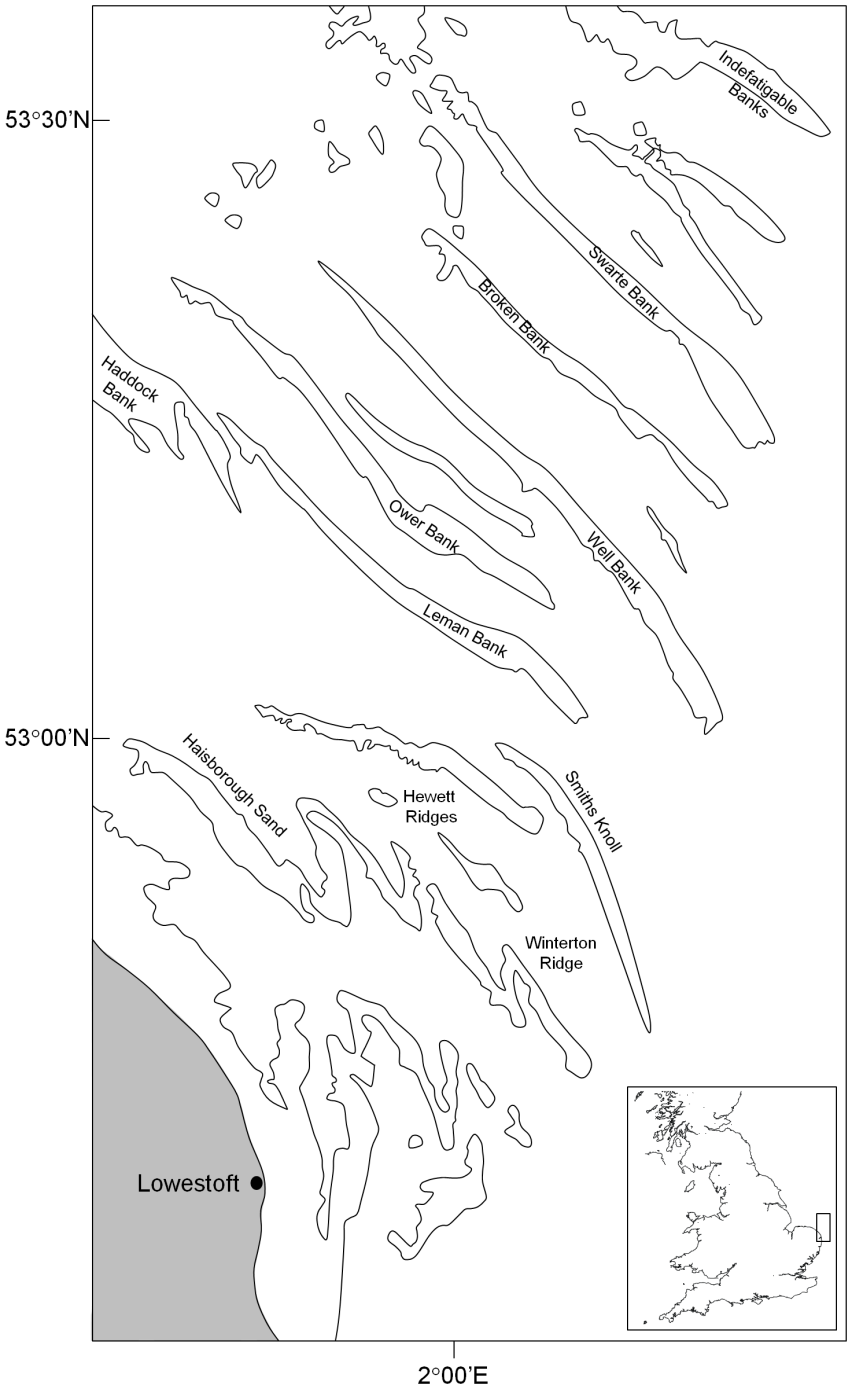
Location of Broken Bank and Swarte Bank in the southern North Sea. Figure adapted from [16].

Broken Bank and Swarte Bank are the outermost of the Norfolk Banks in the southern North Sea. Broken Bank lies 67 km offshore, and has dimensions typical of the tidally-controlled open-shelf linear sandbanks [17] of the outer Norfolk Banks area, at about 30 km long, 1 km wide and with relief of around 30 m above the surrounding sea floor (Fig. 1). Both banks are asymmetric, with a gently sloping SW face and a relatively steep NE face. For the Norfolk Banks in general, based on analyses of past charts and repeat bathymetric surveys, bank migration over decadal and century timescales is generally to the NE, at rates up to 11–15 m yr^−1^ [18], [19], although rates of 40 m yr^−1^ have been recorded [20]. Banks may repeatedly shorten and lengthen, and may migrate SW then NE. For all banks, the bathymetric changes at the head and tail appear high compared to the sideways migration rates [19]. The surface tidal currents in the area of Broken Bank and Swarte Bank are strong, with speeds of 1.6 m s^−1^ and 1.0 m s^−1^ on spring and neap tides, respectively [21]. The bed sediments on and around the banks are loosely-consolidated, well-sorted, fine-grained sands which are transported in an overall clockwise rotation [22]. This transport is thought to take place nominally as bedload, under the influence of tidal currents alone (see also [23]). The addition of wave-induced bed stresses or storm surges can act to increase the transport towards the bank crests and lead to some sand being moved into suspension [22].

### Geophysics

**Figure 2:**
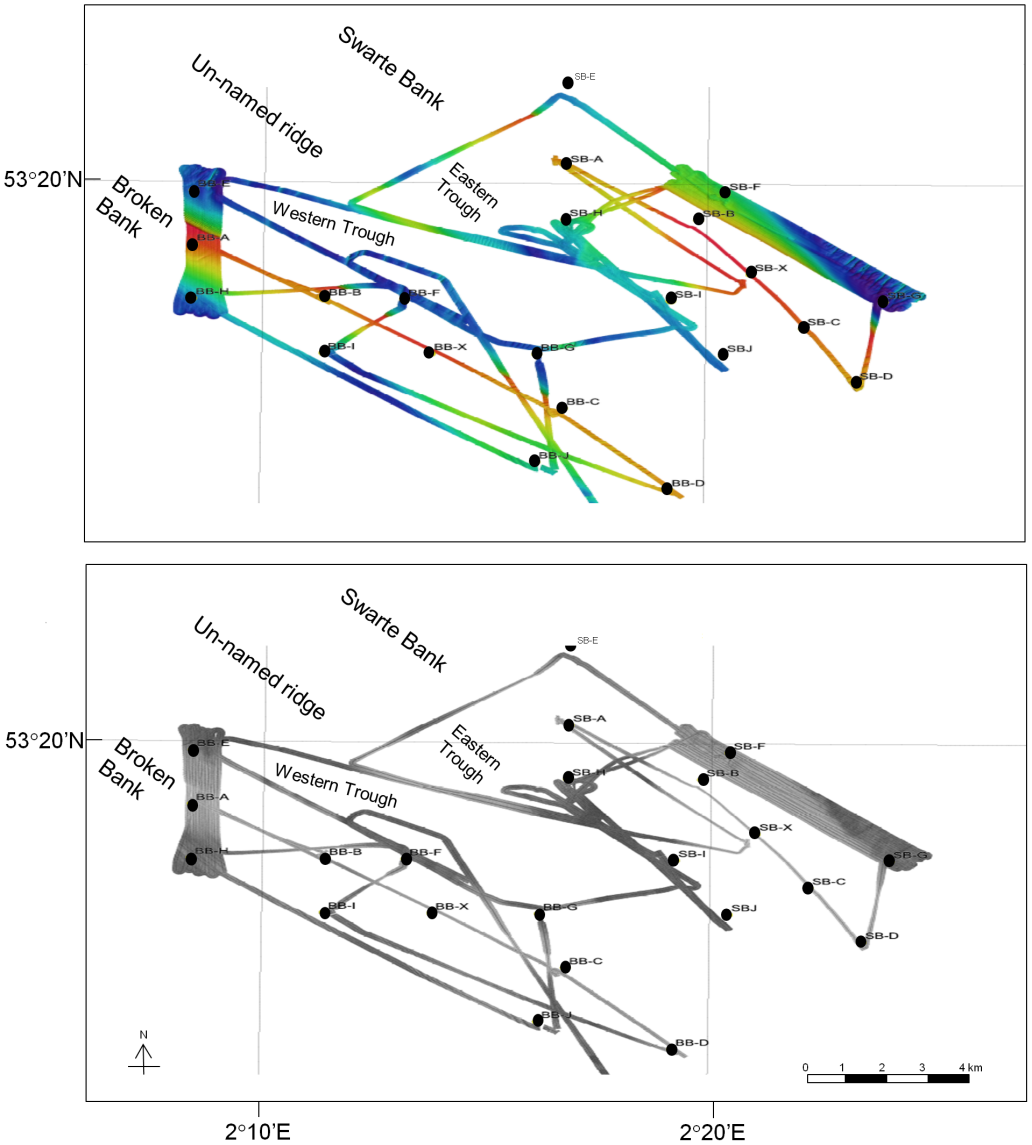
Acoustic survey coverage. The acoustic survey coverage, showing (upper) recorded depth (red  =  shallow, dark blue  =  deep) and (lower) acoustic backscatter, where darker areas indicate greater bed roughness. Sampling stations are also shown. BB  =  Broken Bank, SB  =  Swarte Bank.

**Figure 3:**
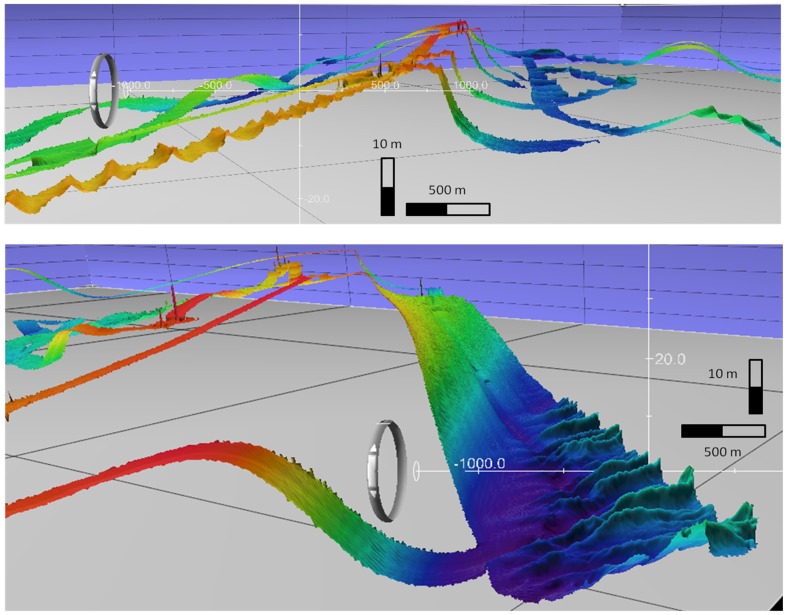
Bathymetry of the surveyed area. Oblique colour-contoured bathymetry of the surveyed area. Vertical exaggeration×35. Black grid squares 5 km apart. Scale bars refer to the intersection point of the white axes. Upper - View towards NW of the asymmetrical cross-section of Broken Bank (sharp crestline in centre) and the low rounded crest of the Un-named ridge (right). Note large asymmetric sandwaves migrating up the SE stoss of Broken Bank (bottom left; details in Table 4). Lower - View towards the NNW of the megarippled lee slope (bedform group v; centre) of Swarte Bank, with field of large asymmetric sandwaves (bedform group i) in the trough migrating to the SE (right; details in Table 4).

In April 2006, the southern portions of Broken Bank and Swarte Bank were surveyed using RV Cefas Endeavour to run a Kongsberg EM3000D multi-beam echo-sounder to provide high-resolution bathymetric data, and a Benthos 1500 digital side-scan sonar to measure seabed acoustic reflectance, which can indicate substrate hardness [11]. The acoustic survey was carried out in parallel to a multi-disciplinary sampling programme designed to provide a general description of the benthic fauna (including meiofauna, macrofauna, epifauna and fish) associated with Broken Bank and Swarte Bank [16]. Individual swath widths covered approximately 150 m on the seabed, and survey lines were designed to run parallel and perpendicular to the overall NW-SE alignment of the bathymetric features. More complete coverage was performed in selected areas of sedimentary interest, including a N-S strip 3×1 km across the SW face, crest and NE face of Broken Bank, and an area 500–800 m wide extending for 7 km along the NE flank of Swarte Bank (Fig. 2 and 3). The survey included coverage of the swale between the banks and an extended swath line off Broken Bank to the SSE. The data were processed using Fledermaus to drape the side-scan images onto the measured bathymetry, with tidal corrections applied. The morphology of the banks and their associated sedimentary bedforms were measured on screen using the Fledermaus viewing software (‘iview4D’), leading to the delineation of a series of areas defined by their sedimentary morphology (bedform groups).

### Sediments

Sediments from both Broken Bank and Swarte Bank were sampled by means of a 1 m^2^ Day grab (Fig. 2; Table 1). Four samples each were collected at five stations along the crest of each sandbank (X, A, B, C, D) and at single sites either side of the sandbanks (F, I). Single samples were collected at four other stations located on the deeper surrounding seabed of each sandbank (E, G, H, J). From each grab sample, two sub-samples were collected with a Perspex corer (3.6 cm diameter, 10 cm^2^ surface area) to a depth of 5 cm, one for the analysis of grain size distribution and one for the study of meiofaunal nematodes. The location of sampling stations was largely dictated by the seabed topography because sufficient draught was necessary under the vessel to allow the collection of sediment samples.

**Table 1:** Positions of the 22 stations sampled in the southern North Sea.

Swarte Bank			Broken Bank		
Station	Latitude	Longitude	Station	Latitude	Longitude
SB-X	53.30 N	2.35 E	BB-X	53.27 N	2.23 E
SB-A	53.34 N	2.28 E	BB-A	53.31 N	2.14 E
SB-B	53.32 N	2.33 E	BB-B	53.29 N	2.19 E
SB-C	53.28 N	2.37 E	BB-C	53.25 N	2.28 E
SB-D	53.26 N	2.39 E	BB-D	53.22 N	2.32 E
SB-E	53.37 N	2.28 E	BB-E	53.33 N	2.14 E
SB-F	53.33 N	2.34 E	BB-F	53.29 N	2.22 E
SB-G	53.29 N	2.40 E	BB-G	53.27 N	2.27 E
SB-H	53.32 N	2.28 E	BB-H	53.29 N	2.14 E
SB-I	53.29 N	2.32 E	BB-I	53.27 N	2.19 E
SBJ	53.27 N	2.34 E	BB-J	53.23 N	2.27 E

Sub-samples for grain size analysis were combined by station and frozen at −20°C prior to analysis. After thawing, the 22 sediment samples were wet sieved through a 500 µm sieve, and the fraction> 500 µm was oven dried at 90°C for 24 h. This fraction was then dry sieved at 0.5 phi intervals, down to 1 phi (500 µm). The fraction <500 µm was freeze dried and analysed on a Malvern Mastersizer S laser particle sizer. The sieve and laser sizer data were merged to form one uniform distribution spanning the size range 0.49 µm −22 mm. It is recognised that freezing samples can alter the grain size distribution, especially of clay-sized grains, but microscope and size analysis both indicated that no clay-sized grains were present in any samples, consistent with previous findings [22], and all samples were treated identically.

Grain size data were grouped using the computer programme ENTROPY [24]. Entropy analysis has been widely used to analyse grain size distributions, avoiding the use of simplified and often inaccurate measures of the shape of the distribution (such as the mean, skewness and standard deviation). Details of the data processing technique have been described in numerous previous studies [24], [25], [26], [27], [28], [29] and are not repeated here. The analysis was used to group the data into a number of grain size groups, ranging in number from two (over-simplified) to 12 (over-complicated) inclusive, the spatial distributions of which were assessed visually. The choice of the number of groups to use in subsequent interpretations is a compromise between achieving a high R(s) statistic (i.e. statistically explaining more of the data; [30]) and accommodating empirical observations within a sedimentologically reasonable model of sediment distribution (e.g. [24]).

Distribution patterns of the various sedimentary bedforms and the grain size data were used together to delineate a series of sedimentary facies for the study area. In the geological literature, there are a number of ways that the term facies has been used [31], including regarding a rock's measured distinctive lithological and/or biological characteristics, as well as the interpreted sedimentary processes and/or sedimentary environment. As used here, the term sedimentary facies means those distinctive physical characteristics of the sediments that occur under particular conditions of sedimentation, reflecting the sum of sedimentary and depositional processes and the environment (e.g. [31]) and which act to distinguish them from adjacent deposits. Geologists have long used fossil evidence of the associated biology as an integral part of defining sedimentary facies.

### Meiofauna

All faunal samples were fixed in 5% formaldehyde in filtered (63 µm) sea water. After washing the meiofauna samples onto a 63 µm sieve, the fauna from all replicate samples were extracted by floatation in Ludox 40 [32]. The extraction was repeated three times before the extracted material was evaporated slowly in anhydrous glycerol and mounted on slides for identification and counting.

Nematodes were identified to genus or species level, using taxonomic keys based on a phylogenetic classification scheme [33], [34], [35], [36]. Species and genera were then assigned to a number of biological traits, i.e. a number of morphological and life-history characteristics, thought or known to represent an important ecological function [37]. Here, six traits were included: mouth morphology, tail shape, pattern of the body wall (cuticle), body size, body shape and life-history strategy (Table 2). There were a total of 25 categories of these traits. A matrix of these biological traits was constructed by assigning to each nematode species/genus its affinity to each trait category.

**Table 2:** Morphological characteristics and life-history of nematode species and genera.

Functional group	Functional importance	Trait categories
Mouth morphology [38]	The mouth cavity shows great diversity in form and reflects the food ingested by the nematodes. It can thus be a good indicator of a species' feeding strategy. Feeding type groupings are fundamental to carbon and energy fluxes through ecosystems, and are linked to nutrient cycling.	Selective deposit feeder, non-selective deposit feeder, epistrate feeder, predator
Tail shape [39], [40], [41]	The tail shape is important in locomotion and reproduction. Long, filiform tails, for example, are considered a special adaptation to fine sand and muddy sediments where only an incomplete interstitial system exists. In these sediments, the tail enables animals to retract from dead-end interstitial passageways that are too narrow to allow the worm to turn around and escape.	Short/round, conical, clavate, elongated/filiform
Pattern of the body wall [42], [43]	The body wall (cuticle) is used as a support and leverage point for movement. It protects the body from drying out and other harsh environments. Nematodes with elaborate cuticular ornamentation tend to be associated with coarser, silt-free sediments, which may be correlated both with their mode of locomotion and with the need for mechanical protection in unstable substrata.	Smooth, annulated, striated, rows of dots/punctuated, rows of structures, with desmen
Body size and shape [44], [45], [46]	Body size and shape affect physiological and ecological features of populations, including metabolic rates, tolerance to chemicals and anoxia, ability to move or migrate, vulnerability to predation etc. Slender nematodes are able to move swiftly through the sediment, but are vulnerable to predation. Predation pressure on stout species may be reduced but so is mobility.	Body size: <1 mm, 1 – 2 mm, 2 – 4 mm,> 4 mm. Body shape: Long/thin, slender, stout
Life-history strategy [47], [48], [49]	Many small species have short generation times of usually about one month or less with high reproduction rates. This largely r-selected life-history strategy is in contrast to the longer life-cycles and fewer offspring of more K-selected species. Some of these often larger species have an annual reproductive cycle.	Coloniser (largely r-selected), intermediate (2 categories), persister (largely K-selected)

Functional groups were chosen because they have been shown or inferred to be functionally important and are thus potentially ecologically meaningful (detailed in [37]). Terminology adapted from [34].

Species and trait diversity and dominance in each sample were expressed as Hill numbers N_1_ and N_2_ [50], respectively. These evenness measures describe different aspects of the community and differ only in their tendency to include or ignore the relatively rare species or traits. Bartlett's and Cochran's tests were used to test for homogeneity of variance before facies-specific differences in species and trait diversity, as well as the relative abundance of functional groups (Table 2) were explored using a one-way analysis of variance (ANOVA). Following the detection of significant differences (P ≤ 0.05) between facies, the Tukey HSD multiple comparisons test was used.

Non-metric multi-dimensional scaling (MDS) ordination using the Bray-Curtis similarity measure was applied to relative abundance data to compare spatial patterns in the taxonomic (i.e. species) and functional (i.e. traits) composition of nematode communities amongst the putative sedimentary facies. One-way analysis of similarities (ANOSIM), the multivariate analogue of analysis of variance, was used to test for significant taxonomic and functional differences between sedimentary facies. Similarity percentages (SIMPER) were calculated to identify the species and traits which both characterised *a priori* sedimentary facies and distinguished between them. The dissimilarity between faunal distribution patterns recorded in the sedimentary facies was computed and an average calculated. We then calculated the contribution of each species and trait to the average similarity within each sedimentary facies and the average dissimilarity between facies. All statistical analyses were performed using the software packages Statgraphics Plus version 3.3 and Primer version 6.1.5 [51].

### Disturbance versus mobility

A variety of publications have used the term disturbance to cover a wide range of anthropogenic and natural sediment transport and mixing processes including dredging, dredge material emplacement, trawling and natural sediment mobility [52], [53], [54]. In our study, we are dealing specifically and purely with the natural movement of sediment, and hence use sediment mobility for maximum clarity. Where we use the term disturbance, it generally refers to the disturbance experienced by organisms during a period of seabed mobility.

## Results

### Sandbank form and superposed bedforms

The banks' gross morphologies include measured lee slopes of nearly 5° (Table 3). Superposed (flow-normal) bedforms include a wide variety of megaripples and sandwaves, of different planform, full-beddedness, size and symmetry (orientation). Some bedforms occur in extensive bedform fields and others in isolation (see [55] for explanation of terminology). Superposed in many areas on both sandbanks are smaller megaripples, which, where resolved, have a steep morphology, indicating that they are probably active. In contrast, some areas between Broken Bank and Swarte Bank and areas associated with their lower stoss flanks contain fields of longitudinal furrows, whose formative processes [56] indicate that these will only be active episodically.

**Table 3:** Morphological characteristics of the positive relief features forming Broken Bank and Swarte Bank.

	Length	Max. width	Max. relief above surrounding seafloor	Water depth at sandbank crest	Max. stoss^a^ slope (in surveyed area)		Max. lee slope (in surveyed area)	
	km	km	m	m	degrees	m/100m	degrees	m/100 m
Broken Bank	33	1.1	30	13–16	1.3	2.2	4.9	8.5
Swarte Bank	37	1.4	28	12–15	0.6	1.1	4.0	7.0

a Many mobile sandy sedimentary bedforms are asymmetrical in vertical cross-section. For tidal sandbanks, sand may be transported over the bedforms in different directions at different times, and may circulate around it in the long term. Nonetheless, there is often a long-term net direction of movement of the bedform, indicated by its asymmetry. Sand is generally transported (obliquely) up the longer shallower stoss slope, over the crest and down the shorter steeper lee slope, which faces the overall direction of movement of the sandbank.

**Figure 4:**
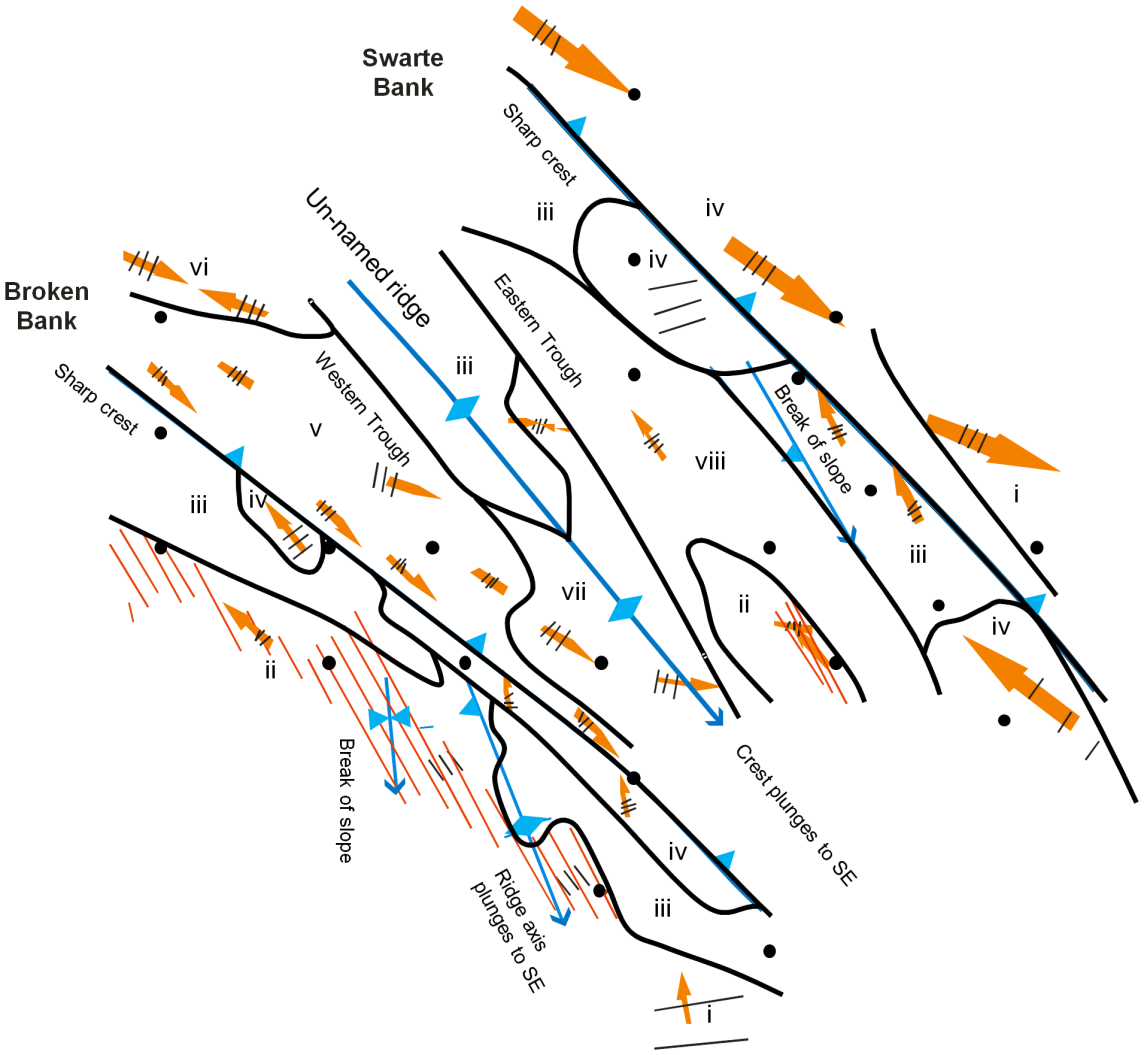
Bedforms in the study area. The 22 stations (black dots) fall into eight broad bedform groups (numbered i – viii; see Table 4), differing in the nature of contained grain size groups (see Table 5). Note - all blue arrows point downhill, indicating the orientation of a significant bed slope. Orange arrows indicate inferred bedform transport direction, with superposed black lines indicating orientation of sandwave crests and sandwave spacing: close lines  =  <100 m; medium  =  100 – 300 m; far apart  = > 300 m. Narrow parallel orange lines indicate areas and orientation of longitudinal furrows.

The bedforms (numbered i – viii) superposed on and between the sandbanks vary across the survey area in a relatively ordered fashion (Fig. 4; detail in Table 4). On the upper stoss flanks (i.e. the SW side), the banks themselves have few clear sedimentary bedforms and show only low relief (<0.5 m; bedform group iii), with, in places, trains of solitary or isolated large sandwaves (bedform group iv). The sandbank lee slopes exhibit very extensive fields of megaripples (bedform group v), with bedform asymmetries indicating bedload sediment transport to the SE. There are also smaller patches of large sandwaves, which are either symmetrical or indicative of net bedload transport to the SE (bedform groups i, vi and vii). The swales between the banks, and areas low on the stoss flanks have a relatively rough topography, which includes areas of longitudinal furrows (bedform group ii) and fields of undulating megaripples (bedform group viii) which occur superposed on the furrows in places. Here, the net bedload transport inferred from the megaripples is generally to the NW.

**Table 4:** Summary characteristics of main bedform groups.

Bedform group	Location	Principal bedforms	Orientation/Asymmetry (degrees)	Size			Description
				Length or spacing	Height or depth	Width or crest length	
Broken Bank							
i	South of Broken Bank	Very large sandwaves	080 – 090, lee to N	∼ 600 m	<5 m	> 250 m	Two full-bedded, straight-crested very large sandwaves, with sandwaves in their troughs.
ii	At and beyond the western edge of Broken Bank	Longitudinal furrows overprinted by full-bedded sandwaves	Furrows − 325–330 Sandwaves − 040, lee to NW	Spacing 80 – 180 m	0.3 – 0.5 m	> 2 km long	Longitudinal furrows with superposed sandwaves.
iii	Upper stoss and crest						Bedforms poorly defined, low relief (<0.5 m).
iv	Central and southern crest	Sandwaves	060 – 090, lee to NW	80 – 100 m	2 – 3 m	> 500 m	Highly asymmetric, full-bedded straight crested sandwaves.
v	Lee slope	Sandwaves	Lee to SE	<40 m	<1.5 m on upper slope		Extensive and uniform field of full-bedded straight-wavy crested sandwaves.
Western Trough							
vi	Lee slope of Broken Bank	Large sandwaves	020 (convergence in the north)	100 – 300 m	> 5 m	Unclear	Complex large relief, including asymmetric straight-wavy crested sandwaves.
v	Western Trough	Small sandwaves	020 – 050	10 – 20 m	<0.3 m	Unclear	Symmetry unclear.
vii	Northern part of Western Trough	Sandwaves	Lee to SE	120 – 150 m	<0.5 m	> 200 m	Wavy-crested.
Un-named ridge							
vii	Trough and southern lee slope	Large sandwaves	350 – 020, lee to E	150 m	<2 m	> 250 m	Asymmetric straight-crested sandwaves.
vii	Lower E slope of un-named bank	Sandwaves	005 – 015 (convergence)	80 – 110 m	<1 m	> 300 m	Asymmetric straight-wavy crested sandwaves.
Eastern Trough							
viii	Broad swale west of Swarte Bank and central stoss slope of Broken Bank	Small sandwaves	050 – 070, some have lee to N	∼ 20 m	<0.5 m	> 80> 150 m (some straight crested> 400 m)	Undulating to rough relief, patchy. Centre and S has topography parallel to longitudinal furrows. Discrete fields of straight-wavy crested sandwaves.
Swarte Bank							
iv	NW part of crest (and southern apron)	Large sandwaves	060 – 090, lee to NW	200 – 600 m	2 – 3 m (0.5 − 2 m in E)	> 500 m	Train of five solitary highly asymmetric sandwaves.
v	Lee slope	Sandwaves	Crests 030 – 050, lee to SE	30 – 40 m (smaller at boundary with i)	<1 m on upper slope, <0.4 m on lower slope	> 200 m	Very extensive and uniform field of full-bedded straight-crested sandwaves along the bank's lee slope.
i	South-eastern edge of lee slope	Large sandwaves	030 – 040, lee to SE	50 – 200 m	2 – 6 m	> 800 m	Asymmetric full-bedded, wavy-straight crested (some double-crested). Height increases to the E.
iii	Upper stoss of Swarte Bank				0.3 – 0.5 m		Bedforms poorly defined, low relief (<0.5 m).

The key physical features are therefore that (Fig. 4; Table 4):

1. For the positive relief features of Broken Bank and Swarte Bank, bedform transport is relatively active, with transport of sand to the NW across the stoss slope and along the crest, and to the SE along the lee slope, i.e. in an apparent clockwise circulation (agreeing with [22]).
2. Between the two sandbanks, including the Western Trough, the Un-named ridge and the Eastern Trough, the bed contains a variety of bedforms and a complex distribution, closely associated with the major bathymetric relief.
3. The flat seabed away from the sandbanks is relatively starved of sand, and here fast storm-associated shelf flows have caused seabed erosion in the form of longitudinal furrows. Where superposed by megaripples, the furrows have not recently been active.
4. Overall, the troughs and lower stoss slopes of Broken Bank and Swarte Bank contain coarser sediments and have a rougher seabed, together with bedforms (longitudinal furrows) which indicate episodically fast flows. This is in contrast to the upper stoss where the sediment is finer and where the mostly smoother seabed is inferred to be more continuously mobile.

### Sediment granulometry

**Figure 5:**
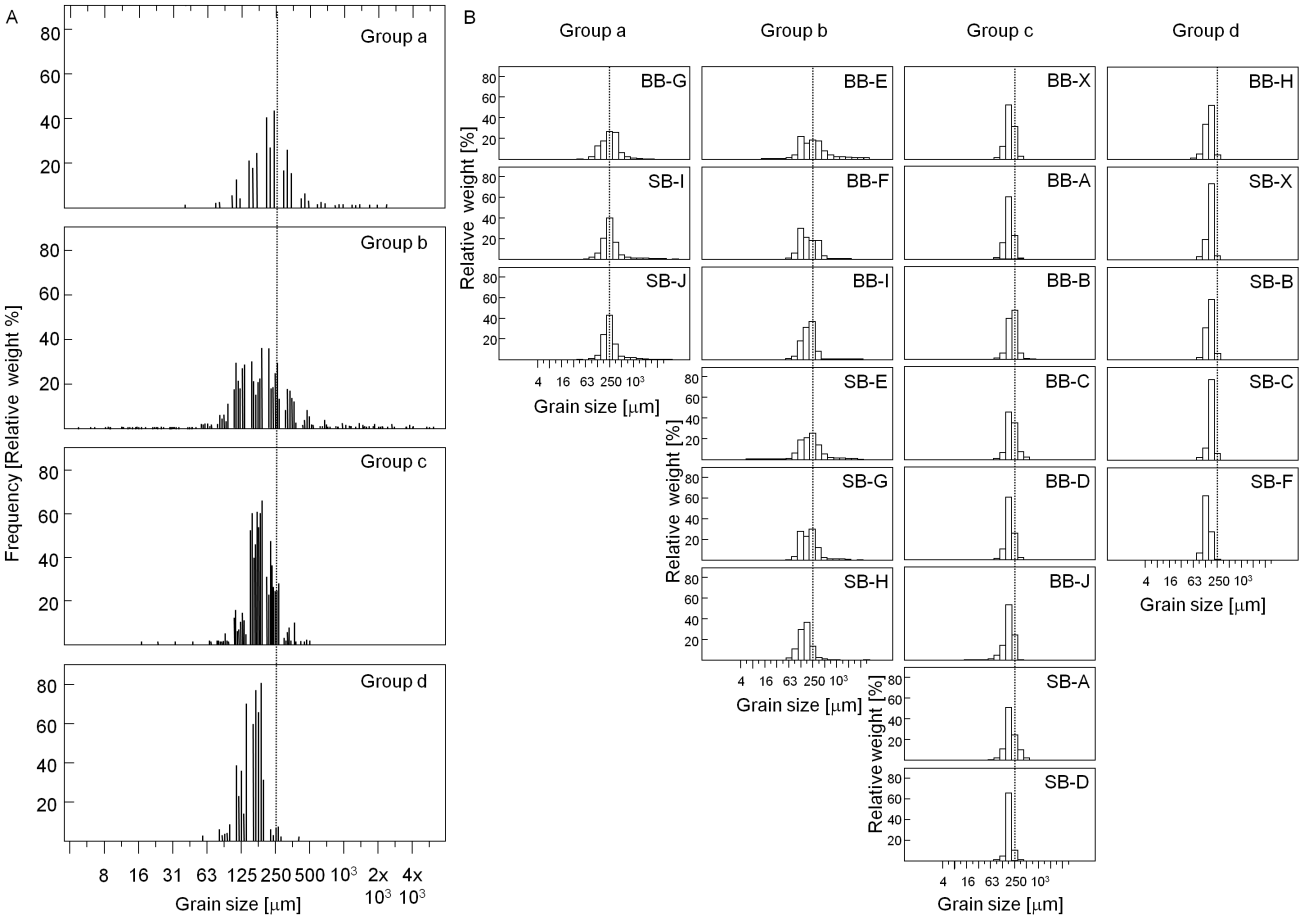
Grain size distribution of all 22 sediment samples. A. All samples of each group, plotted together to illustrate the key differences between the groups. B. All samples within each group. The vertical dotted line at 250 µm helps illustrate the between-group shift in the peak of the grain size distribution. Note that grain size groups a and b have a minor gravel component.

The seabed sediments are mostly fine and medium sands, with a modal grain size of 180 – 250 µm, and with a minor gravel component in some samples. Based on their entire size distributions, the samples fall into four broad grain size groups (numbered a–d; Fig. 5A, B). In relative terms, the slightly gravelly sediments of groups a and b are poorly sorted and tend to occur off the main banks in areas with uneven topography and high acoustic roughness, including in areas close to the northern termini of longitudinal furrows. The groups' characteristics are relatively simple and, in combination with the bathymetric and side-scan datasets, relationships between them are readily interpretable (Table 5). The data are consistent with the finding of [22] that sand transport on the banks themselves will generally occur as bedload, but with suspension of sand likely at times of superposed waves; this may help explain the relatively poorly defined bedforms on the upper stoss of the banks. During severe storms, mobilisation of all the sampled grain sizes is possible.

**Table 5:** Summary characteristics of grain size groups.

Grain size group	Sediment	Main characteristics and water depth	Spatial distribution (i.e. samples forming the group)	Comparison between groups	Interpreted sedimentological processes
a	Slightly gravelly unimodal medium sand	Modal size ∼ 250 µm, with up to 25% fine sand. 34 – 39 m	Samples in the Western Trough (BB-G) and the southern part of the Eastern Trough (SB-I, SB-J).	Coarsest sediments, with less sand than other groups. Sand is medium-grained.	Sand fraction probably mobile on regular basis, over a gravel lag and/or medium sand is being eroded from the seabed.
b	Slightly gravelly fine and medium sand	Poorly sorted fine-medium sand with a minor gravel component. 34 – 40 m	In the troughs between the main banks and low on the main banks' stoss slopes (BB-E, BB-F, BB-I, SB-E, SB-G, SB-H).	Equivalent to group a plus fine sand. All sizes present across all groups.	Mixture of other groups; likely to represent a thin layer of mobile fine sands over surface of group a material.
c	Unimodal fine to medium sand	Modal size ∼ 180 µm, with up to 30% medium sand. 17 – 33 m	Along the crest of Broken Bank (BB-A, BB-B, BB-X, BB-C, BB-D) and Swarte Bank (SB-A), and on the stoss slope of SE Broken Bank (BB-J) and Swarte Bank (SB-D).	Relatively well sorted.	Regular movement of sand with, in calm seas, perhaps only a few tides per fortnight with no movement. Long-term accumulation.
d	Unimodal fine sand	15 – 34 m	On the crest and NE flank of Swarte Bank (SB-F, SB-B, SB-X, SB-C) and one sample on the northern stoss of Broken Bank (BB-H).	Relatively well sorted.	Daily movement of sand within megaripple fields. Long-term accumulation.

### Sedimentary facies

**Figure 6:**
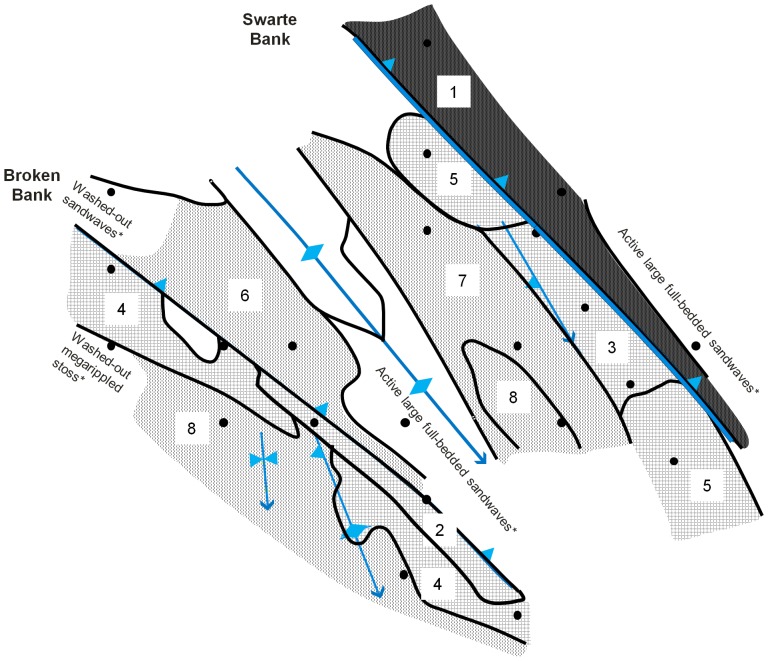
Distribution of sedimentary facies groups in the study area. Facies shadings are grouped according to their relative level of sediment mobility (Table 6). The 22 stations (black dots) fall across a range of sedimentary facies, numbered 1 – 8 (details in the text and in Table 6). Facies with less than three observations (*) were excluded from statistical analyses. For explanation of annotations see Fig. 4.

The survey area comprised a total of 11 sedimentary facies, eight of which (numbered 1 – 8) were included in our statistical analyses (Fig. 6). Facies are primarily based on the physical evidence for the frequency, magnitude and inferred direction of sediment movement, based mostly on bedforms, and the tendency for long-term erosion or accumulation, based on the evidence of exposed coarse substrates. Facies with less than three observations were omitted from our statistical analyses, including ‘Active large full-bedded sandwaves’ at stations BB-G and SB-G (total of two observations), ‘Washed-out sandwaves’ at the lower lee to trough of Broken Bank (single observation at station BB-E) and the ‘Washed-out megarippled stoss’ of Broken Bank (single observation at station BB-H).

The facies names highlight the major features which define each facies and which distinguish them from adjacent deposits (Table 6; Fig. 6). The sedimentary facies delineated for the study area differed with regard to 1) their location across the study area, 2) their observed grain size distribution and seabed topography, and 3) their inferred history, intensity and frequency of sediment mobility. The concept of mobility, as used here, can thus include factors such as duration, magnitude, period since previous mobilisation, time of year, depth, and physical nature (i.e. erosion, transport, accumulation, mixing and unmixing, covering and uncovering of lag surfaces etc.) as well as associated changes in oxidation state and other biogeochemical parameters. With a view towards timescales of around a month, in line with meiofauna turnover times, our eight facies fall into four groups with respect to their relative level of sediment mobility. Facies 1 is termed high, facies 2, 3 and 4 are high-moderate, facies 5 is moderate and facies 6, 7 and 8 are episodic (Table 6).

**Table 6:** Physical features and key meiofaunal characteristics of sedimentary facies.

	Active megaripples at Swarte Bank (1)	Active full-bedded sandwaves at the crest of Broken Bank (2)	Megarippled stoss of Swarte Bank (3)	Washed-out megaripples at the stoss of Broken Bank (4)	Active large solitary sandwaves at the stoss/apron of Swarte Bank (5)	Active large solitary sandwaves in the Western Trough (6)	Washed-out undu-lating bed with sand-wave fields in the Eastern Trough (7)	Sandwaves over longitudinal furrows off the banks (8)
Station(s)	SB-E, SB-F	BB-X, BB-C	SB-X, SB-B, SB-C	BB-A, BB-B, BB-D	SB-A, SB-D	BB-F	SB-H, SB-I	BB-I, BB-J, SB-J
No. of obs.	5	8	12	12	8	4	5	6
GS groups	b, d	c	d	c	c	b	a, b	a, b, c
Bedf. groups	v	iv	iii	iii	iv	v	viii	ii
Rel. level of sediment mobility	High	High-moderate	High-moderate	High-moderate	Moderate	Episodic	Episodic	Episodic
Description (partly interpretative)	Sediments and megaripples mobile on a daily basis, except possibly at neap tides.	Large sandwaves migrate frequently.	Sediments mobile on a daily basis, but megaripples less frequently so.	Sediments mobile on a daily basis, but megaripples less frequently so.	Large sandwaves migrate frequently.	Large sandwaves migrate, superposed by small sandwaves and megaripples. Gravel grains widespread.	Sandy grains regularly mobile across and formed into sandwaves and megaripples at times, perhaps during storms, when widespread gravel grains also mobile.	Furrows formed and reactivated during severe storms, then become inactive for long periods, during which sandwaves become superposed and finer sediment accumulates in furrows.
Sedimentary environment (partly interpretative)	Little spatial variation in surface sediment, and probably also homogenous to a depth of a few decimetres.	Minor gradients in the nature of benthic habitats across the sandwaves. Sediments probably homogenous to a depth of a few decimetres.	Little spatial variation in surface sediment, and homogenous to a depth of a few decimetres.	Little spatial variation in surface sediment, and probably also homogenous to a depth of a few decimetres.	Moderate gradients in the nature of benthic habitats across the sand-waves, although sediments relatively similar; different ha-bitats in the troughs. Variation with depth, especially across the troughs.	Moderate gradients in the nature of benthic habitats across sandwaves; different habitats in the troughs. Patchy degree of variation with depth, especi-ally across the troughs between sandwaves.	Moderate gradients in the nature of benthic habitats. Potential rapid accumulation after storms.	Range of benthic habitats in and between furrows and sandwaves, affected by the time since creation or re-activation and rate of infilling. Significant spatial and depth-related differences in sediment type.
Nematode assemblages	Highly similar nematode communities dominated by contrasting trait groups, comprising small deposit feeders and large predators.	High proportions of nematodes with a heavily cuticularised body wall; a ratio of epistrate feeders vs. non- selective deposit feeders> 1.	Considerable faunal similarity to facies 2; subtle differences in the relative abundance of dominant species and traits.		Varied assemblages encompassing deposit and epistrate feeders of various sizes, body ornamentations and reproductive potential.	Considerable overlap in the species and trait composition with facies 8. Trait similarity between facies 6 and 7 exceeds similarity within the individual facies.		High abundance of the dominant multi-species trait group and a limited range of other trait groups combining a deposit or epistrate-feeding habit with a medium-sized, slender body.

Facies number in brackets. GS  =  grain size.

### Distribution of nematode species and functional traits relative to sedimentary facies

At all stations, nematodes consistently comprised> 81% of the total meiofauna abundance. A total of 9171 nematodes was identified, with abundances per sample ranging from 47 in washed-out megaripples at the stoss off Broken Bank (station BB-D) to 434 in active megaripples at Swarte Bank (station SB-F). Nematode assemblages in the study area were taxonomically and functionally diverse. A total of 118 species was recorded which fell into 77 trait groups.

**Figure 7:**
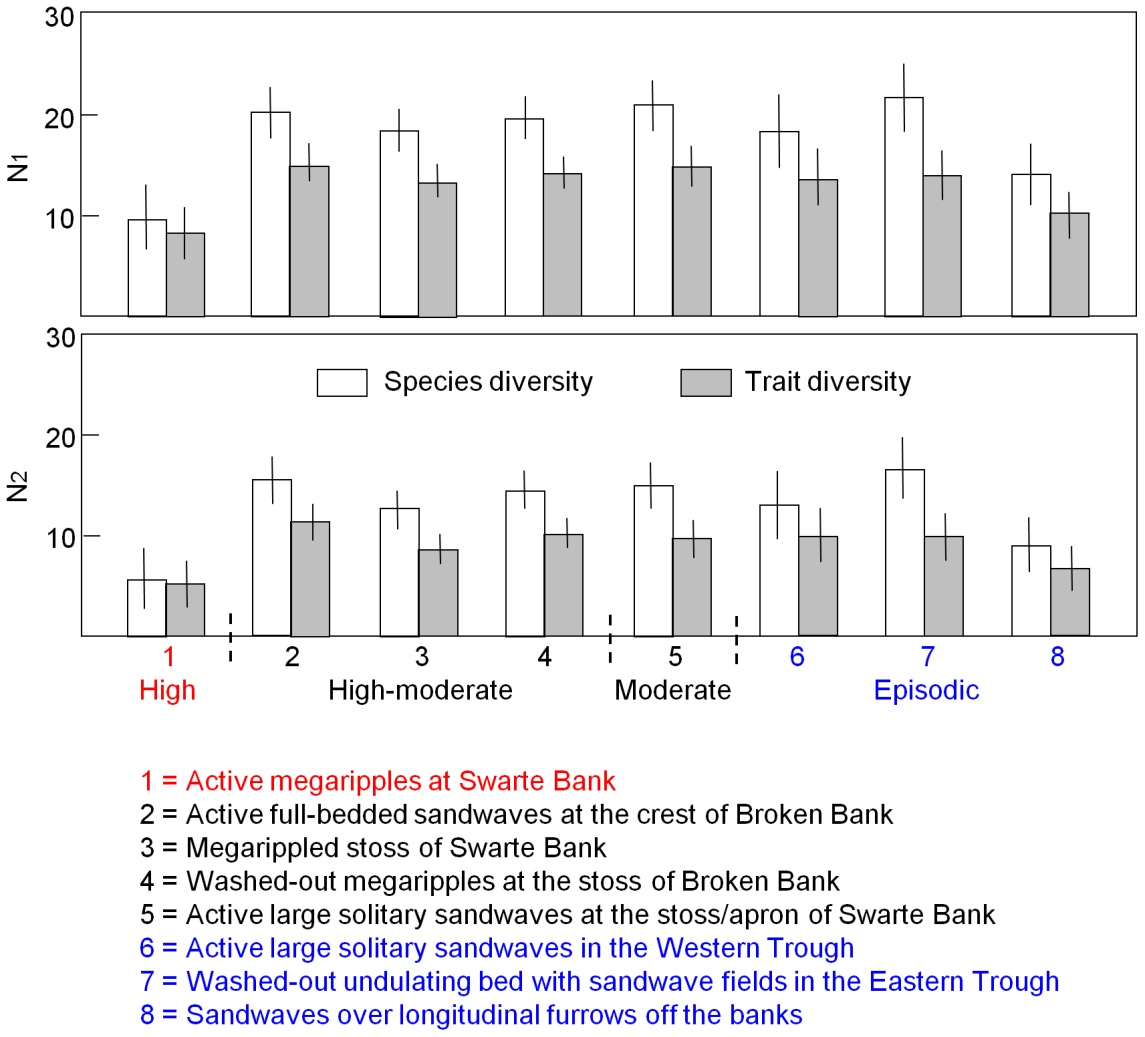
Species and trait diversity of nematode assemblages. Mean (± 95% pooled confidence interval) species and trait diversity of nematode assemblages recorded in eight sedimentary facies coded by relative level of sediment mobility. Diversity is expressed as Hill numbers N_1_ and N_2_.

Results from the one-way ANOVA revealed significant differences between sedimentary facies in terms of taxonomic and trait diversity (N_1(species)_: F = 6.71, P <0.01; N_1(traits)_: F = 4.66, P <0.01) and dominance (N_2(species)_: F = 6.96, P <0.01; N_2(traits)_: F = 3.72, P = 0.02). Taxonomic and trait diversity (N_1_) were significantly lower in facies 1 and 8 (‘Active megaripples at Swarte Bank’ and ‘Sandwaves over longitudinal furrows off the banks’) than in most of the others. High numbers of some dominant species and traits in facies 1 resulted in a low N_2_ value, whereas the low N_2_ value observed in facies 8 was a consequence of the distribution of nematodes across a limited number of species and traits (Fig. 7).

Functionally, nematode assemblages were dominated by epistrate-feeding species that combined fast turnover times and an ornamented, relatively small, slender body (Table 7). A total of five chromadorid and cyatholaimid species fell into this category. Another multi-species trait group, differing from the dominant trait group only in the type of body ornamentation, as well as two single-species trait groups (comprising *Xyala striata* and *Metadesmolaimus pandus*, respectively) were also highly influential in determining taxonomic and functional distribution patterns across sedimentary facies.

**Table 7:** Taxonomic affiliation and morphological/functional characteristics of nematode trait groups which discriminate between sedimentary facies.

	Dominant multi-species trait group	Sub-dominant multi-species trait group	Single-species trait group	Single-species trait group
No. of species	5	3	1 (*Xyala striata*)	1 (*Metadesmolaimus pandus*)
Taxonomic affiliation	Chromadoridae, Cyatholaimidae	Microlaimidae	Xyalidae	Xyalidae
Buccal morphology	Epistrate feeder	Epistrate feeder	Non-selective deposit feeder	Non-selective deposit feeder
Tail shape	Conical	Conical	Conical	Clavate
Cuticle pattern	Rows of dots/punctuated	Striated	Structures	Striated
Body size	1–2 mm	1–2 mm	1 – 2 mm	<1 mm
Body shape	Slender	Slender	Slender	Slender
Life-history strategy	Coloniser	Coloniser	Intermediate	Coloniser

**Figure 8:**
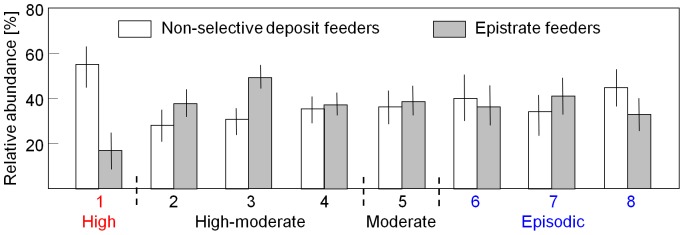
Relative abundance of non-selective deposit feeders and epistrate feeders. Mean (± 95% pooled confidence interval) relative abundance of non-selective deposit feeders and epistrate feeders recorded in eight sedimentary facies coded by relative level of sediment mobility. Facies numbers as in Fig. 7.

**Figure 9:**
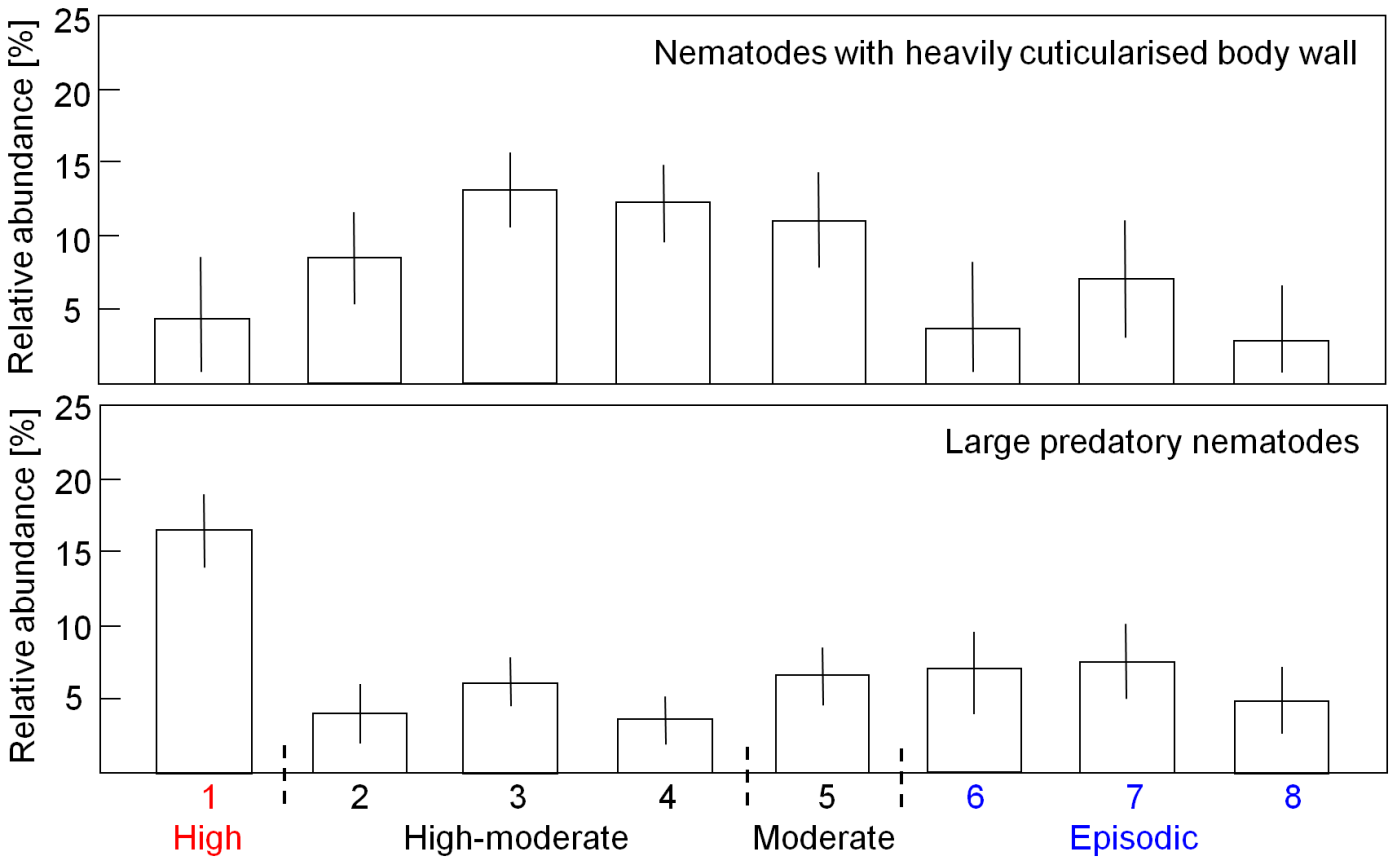
Relative abundance of nematodes exhibiting specific functional traits. Mean (± 95% pooled confidence interval) relative abundance of nematodes exhibiting a heavily cuticularised body wall (top) and mean (± 95% pooled confidence interval) relative abundance of large predators (bottom) recorded in eight sedimentary facies coded by relative level of sediment mobility. Facies numbers as in Fig. 7.

Results from SIMPER and ANOSIM analyses (Tables 6 and 8) indicate that there were differences in nematode community structure between facies. Nematode communities assigned to facies 1 (‘Active megaripples at Swarte Bank’) exhibited relatively high average similarity in taxonomic and functional composition, probably reflecting the relative homogeneity of the sedimentary environment. In contrast to other facies, the communities of facies 1 were taxonomically more similar than functionally, indicating that those species that were unique to individual samples were mostly single-species trait groups. The samples were highly dominated by contrasting trait groups, comprising *Metadesmolaimus pandus* and large predators, respectively. The trait groups differed not only with regard to feeding habit, but also in cuticle pattern (striated versus smooth) and body size (<1 mm versus> 2 mm). The relative abundance of non-selective deposit feeders was significantly higher (F = 4.66, P <0.01) in this facies and that of epistrate feeders significantly lower (F = 7.07, P <0.01) compared to other sedimentary environments (Fig. 8). This was the only sedimentary facies where larger predatory nematodes were present in notable numbers (F = 12.14, P <0.01; bottom of Fig. 9).

**Table 8:** Faunal differences (R-values) between sedimentary facies.

	Active megaripples at Swarte Bank (1)	Active full-bedded sandwaves at the crest of Broken Bank (2)	Megarippled stoss of Swarte Bank (3)	Washed-out mega-ripples at the stoss of Broken Bank (4)	Active large solitary sandwaves at the stoss/apron of Swarte Bank (5)	Active large solitary sandwaves in the Western Trough (6)	Washed-out undulating bed with sandwave fields in the Eastern Trough (7)
Active full-bedded sandwaves at the crest of Broken Bank (2)	**1/0.99**						
Megarippled stoss of Swarte Bank (3)	**1/0.99**	**0.83/0.80**					
Washed-out megaripples at the stoss of Broken Bank (4)	**0.99/0.94**	**1**	**0.54/0.43**				
Active large solitary sandwaves at the stoss/apron of Swarte Bank (5)	**0.99/0.90**	**0.51/0.35**	**0.40/0.47**	0.22/0.03			
Active large solitary sandwaves in the Western Trough (6)	**0.89/0.73**	**0.99/0.90**	**1/0.93**	**0.97/0.75**	**0.97/0.69**		
Washed-out undulating bed with sandwave fields in the Eastern Trough (7)	**0.97/0.88**	**0.78/0.77**	**0.93/0.85**	**0.65/0.51**	**0.70/0.46**	0.68/0.28	
Sandwaves over longitudinal furrows off the banks (8)	**0.50/0.34**	**0.74/0.66**	**0.92/0.87**	**0.78/0.65**	**0.73/0.57**	0.14/−0.18	0.44/0.34

R-values derived from pair-wise analysis of similarities (ANOSIM) tests for differences between sedimentary facies based on relative abundance data of species/functional traits. Bold indicates significant difference at P <0.01. Facies number in brackets.

A high proportion of nematodes with a heavily cuticularised body wall was a common and significant functional feature of facies 2 (‘Active full-bedded sandwaves at the crest of Broken Bank’; top of Fig. 9) as well as other hydrodynamically active facies containing either megaripples (e.g. facies 3 and 4) or sandwaves (e.g. facies 5). In this group of facies, the proportion of epistrate feeders tended to be higher than the relative abundance of non-selective deposit feeders (Fig. 8; Table 6). The species- and trait-rich facies 5 (‘Active large solitary sandwaves at the stoss/apron of Swarte Bank’) was associated with the most taxonomically and functionally varied assemblages. The dominant trait groups differed little from those encountered in facies 8, but their relative abundance was notably different. In facies 5, the proportion of *Metadesmolaimus pandus* was lower, with associated higher relative abundance of heavily ornamented nematodes such as *Xyala striata* (top of Fig. 9). This facies comprises a range of sedimentary environments, which are correspondingly colonised by functionally diverse assemblages (Table 6). These assemblages encompassed deposit and epistrate feeders of various sizes (<1 – 2 mm), body ornamentations, ranging from moderately (i.e. striated or punctuated) to heavily (i.e. annulated or structured) cuticularised, and varied reproductive potential (coloniser to intermediate).

*Metadesmolaimus pandus* (23% of all nematodes) and the dominant multi-species trait group (24% of all nematodes) were abundant in facies 8, (‘Sandwaves over longitudinal furrows’) as was a limited range of other trait groups which combined a deposit- or epistrate-feeding habit with a striated, medium-sized (1 – 2 mm), slender body. Nematode assemblages in this facies exhibited a relatively high similarity with those recorded in facies 6 and 7 (Tables 6 and 8).

From the above, the key faunal features are that:

1. Taxonomically and functionally distinct nematode communities were present in facies 1 and 5, characterised by low and high faunal diversity, respectively (Table 6).
2. There was a considerable overlap in the species and trait composition of nematode assemblages collected in facies 2, 3 and 4. Heavily ornamented nematodes were common here and numbers of epistrate feeders generally exceeded that of other feeding types.
3. Some sedimentary environments located in the Western Trough and Eastern Trough and off the banks (i.e. facies 6, 7 and 8) differed little with regard to their taxonomic and functional structure. Small and medium-sized deposit- or epistrate-feeding species dominated nematode assemblages.

**Figure 10:**
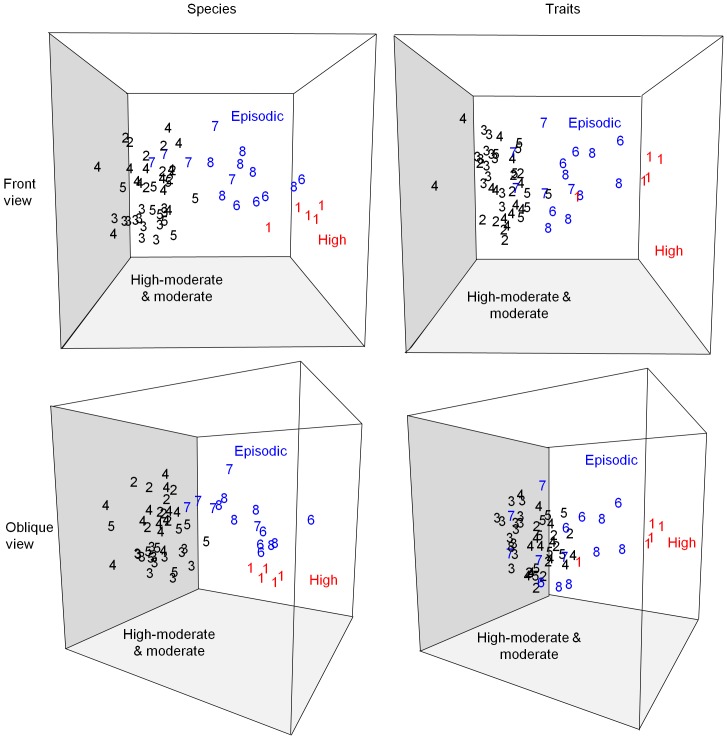
Non-parametric multi-dimensional scaling (MDS) ordination of nematode assemblages. 3D-view of non-parametric MDS ordination of nematode assemblages coded by facies (numbered as in Table 6 and Fig. 7) and relative level of sediment mobility (see text for details). The plots are derived from a similarity matrix of relative abundance of species (left) and traits (right).

These key features tend to indicate a range of faunal similarities between sets of facies, which led to readily interpretable patterns in the MDS ordination (Fig. 10). Distinct nematode communities were present in active megaripples at Swarte Bank (facies 1, termed high mobility), where spatially homogeneous bedforms are mobilised daily by tides. These communities form a tight cluster at the bottom right hand side of the ordination plots. Nematode assemblages from environments where sediment mobility was more intense yet infrequent or absent on timescales of a month (facies 6 – 8, termed episodic mobility) form a broad cluster across the centre and upper part of the ordination plots. This is consistent with the variable suite of sedimentary environments. Data from facies 8 are particularly widely spaced on all plots. Sampling stations where nematodes experience high to moderate intensities and frequencies of sediment mobility (facies 2 – 5, termed high-moderate and moderate mobility) form a medium-sized cluster, within which most facies overlap except for facies 3, representing relatively active megaripples, which forms a separate cluster.

## Discussion

Interstitial habitats such as those occupied by meiofauna are broadly distributed across the planet, and the complexity of the associated physical sedimentary environments has been present throughout the evolutionary history of meiofauna (e.g. [57]). Hence, it is clear that processes operating in the geological past have shaped what we sample and see today. A key relevant timescale is the last few thousand years, when the southern North Sea was inundated by the rising Post-Glacial sea [58] and became subject to marine processes [59]. At shorter timescales, the periods between major storms and the various tidal cycles became relevant to the benthic biology. Put simply, the benthic fauna we sample depends in part on the day-to-day modern processes but also on the episodic storm-associated processes, and the history of both.

A variety of sediment transport processes in our survey area, operating at different magnitudes and frequencies, created a wide range of sedimentary environments for meiofaunal nematodes (Table 6). The presence of active sedimentary bedforms, such as sandwaves (i.e. crest spacing of 10s – 100s m) and megaripples (i.e. crest spacing of 1s – 10s m), for example, have important consequences for the benthic ecology of sandbanks. Sandwave and megaripple crests tend to have different sediments, sometimes subtly, than their troughs [60], [61], [62], [63], providing a variety of different niches for colonising meiofauna. Previously published review articles on the effects of physical seabed disturbance on benthic ecosystems [64], [65] suggest that species-specific properties such as population growth rates and tolerance to environmental change affect a species' susceptibility to disturbance as well as its recovery from it. However, only recently have scientists begun to consider the key role of the sedimentary regime in determining the pre-disturbance faunal distribution patterns and as a factor in the post-disturbance phase of ‘recovery’ [66], [67].

### Benefits of the sedimentary facies approach

Delineating and using sedimentary facies can help to better understand the way in which the local complexity of the seabed might determine the number and type of available niches for meiofaunal nematode communities. In our context, sedimentary facies inherently include the key features of the bedforms and sediments themselves (e.g. [31]), thus incorporating many aspects of sediment mobility and the tendency for erosion or accumulation, which vary across environments. Therefore, sedimentary facies tend to group various combinations of physical and time-variable factors, improving the likelihood of forming meaningful and readily interpretable relationships with the infaunal data.

**Figure 11:**
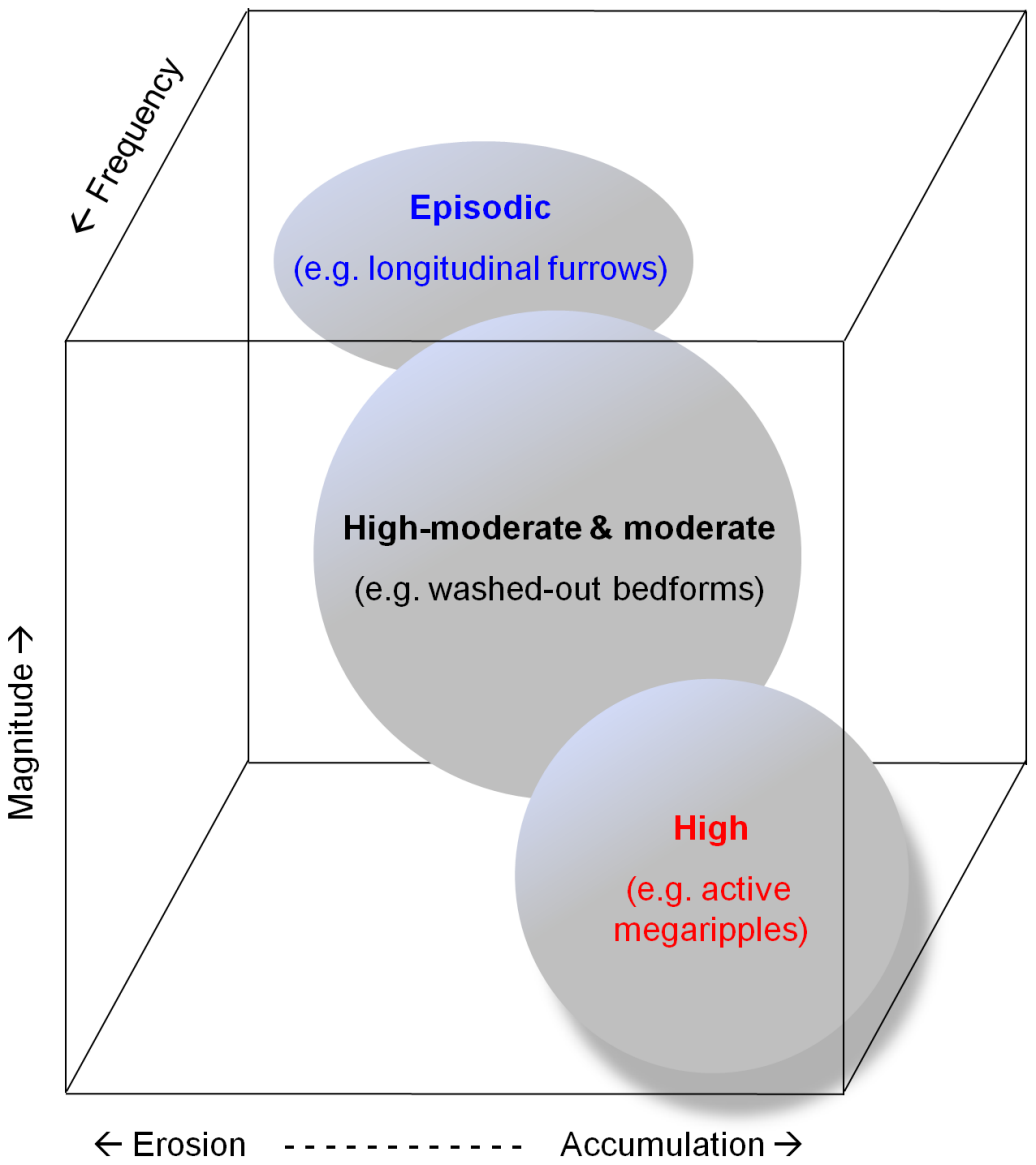
Conceptual diagram of three key controls on sedimentation in the study area. General relative locations of groups of sedimentary facies representing high, high-moderate and moderate, and episodic sediment mobility, plotted against the frequency of sediment mobility, its magnitude, and the continuum between erosion, translation (i.e. sediment throughput) and sediment accumulation.

With an emphasis on the sandy facies appropriate to our study area, the general relationships between the groups of sedimentary facies representing high, high-moderate and moderate, and episodic sediment mobility can be illustrated by using three key parameters: the frequency of sediment mobility, its magnitude, and the continuum between erosion, translation (i.e. sediment throughput) and sediment accumulation (Fig. 11). Our facies groups fall into different areas of this diagram. This diagram helps visualise some of the concepts of our data and its interpretation, and we hope helps others in designing measurement and monitoring programmes, and interpreting collected data.

### High mobility

A common assumption of ecological theories addressing the causes of faunal variability in space and time is that species or functional groups with different morphology, physiology or behaviour dominate under different environmental regimes [68], [69], [70], [71] and this is consistent with our findings. In facies 1 (‘Active megaripples at Swarte Bank’), for example, daily movement of sediments and bedforms will most likely have led to the resuspension, burial and/or mechanical damage of microalgal cells [72]. It is thus logical to find that epistrate feeders, whilst abundant elsewhere on the sandbanks, were less numerous here. We hypothesise that organic material accumulates preferentially in megaripple troughs at slack tide, some of which is subsequently buried by migrating ripple crests during succeeding tidal flows, a recognised sedimentary process [60] which has been postulated as important for meiofauna on intertidal sandflats (e.g. [73]). This subsequently attracts deposit-feeding nematodes from surrounding areas such as the small-sized *Metadesmolaimus pandus* who, together with a contrasting trait group comprising large predatory nematodes (including *Viscosia*), dominated colonist communities. These disturbance-resistant nematodes [74] were most efficient in exploiting available food sources.

### High-moderate mobility

Larger sedimentary bedforms comprising small sandwaves and washed-out megaripples are located along the crests of both banks (facies 2 – 4) and indicate relatively high magnitudes and frequencies of bed sediment transport. However, in contrast to facies 1, the bedforms may persist for longer (i.e. weeks to years as opposed to hours to days) and be more spatially varied. Accordingly, we recorded a range of coexisting epistrate feeders and sub-dominant deposit feeders. As has been shown for other dynamic sedimentary environments [75], [76], much of the available space was filled by competitors most resistant to physical damage, in our case species with a heavily cuticularised body wall.

### Moderate mobility

Solitary sandwaves on the lower stoss and apron of Swarte Bank (facies 5) were characterised by moderate spatial gradients in the benthic habitat across and along the bedform (Tables 4 and 6). Compared to smaller, more frequently active bedforms (i.e. megaripples and smaller sandwaves, see above), the physical conditions here appear less critical in controlling the success or failure of species and functional groups. Biological stress such as intense competition for food and space is likely to be gradually mediated through biological interactions (see review by [77], [78]). Because different functional types use different resources, use the same resources in different ways or use the environment in a way that allows them to reduce or avoid competition with other functional types [79], the relatively high environmental heterogeneity increased the number of microhabitats for nematodes. The functional types recorded in our samples showed responses indicative of the trade-offs between resistance to disturbance, environmental tolerance and competitive abilities. Deposit and epistrate feeders of various sizes, body ornamentations and reproductive potential coexisted in taxonomically and functionally diverse colonist communities.

### Episodic mobility

The relatively low elevation seabed between Broken Bank and Swarte Bank comprised sandwave fields of varying topography and extent (e.g. facies 6 and 7). In common with facies 8, at the steep lee slope of Swarte Bank, these areas of seabed probably undergo repeated and complex ecological transitions, associated with the irregular timing and nature of seabed mobilisation (e.g. as a result of storms in the study area). The initiation and episodic regeneration of bedforms (including longitudinal furrows) by strong storm-associated flows is related to very high rates of sediment transport, involving largely passive transport of meiofauna, and at these times, much of the study area would constitute a solely physically driven benthic system (sensu [80]). However, between such events, the area occupied by facies 6 – 8 would experience longer periods of being a more biologically influenced system, geologically characterised by the inter-storm formation of superposed sandwaves and infilling of the furrows, and where nematode communities respond to both the varied sedimentary environment but also to various biological interactions.

Such an episodically influenced change in seabed character probably also extends to those facies located North and West of the furrowed areas, away from the SE tips of the banks. It is likely that, especially during periods of decreasing sediment transport associated with the decaying phase of storms, a phase of relatively high net sediment accumulation occurs here over a few hours, and perhaps up to a day or so, depending upon the particular storm's characteristics. This phase of accumulation would be followed by an extended period where colonisation and assemblage development will take place, associated with the ambient, strongly tidal, oceanographic regime. Resident nematode communities appear to reflect such sedimentary complexity. In our particular case, the dominance of small- to medium-sized deposit- and epistrate-feeders of high reproductive potential most likely represents communities at a relatively early post-mobility stage.

### Implications for future studies

Taxonomically and functionally disparate communities inhabiting the sedimentary facies defined in our survey area can be considered a complex product of the various sedimentary contrasts inherent within different sedimentary facies and the general sedimentary nature of the area being considered. Regarding variation within facies, the sedimentary environments experienced by meiofauna will be distinctly different across the variety of sub-environments across a sandwave-dominated facies, where troughs, stoss slopes, crests and lee slopes are dynamically and texturally different. There will also be great differences in those facies where longitudinal furrows occur with differing levels of infill. Further, sandwaves are much larger physical features than megaripples, and therefore their rates of horizontal migration and morphological change tend to be much slower. In our study area, sandwaves also tend to be much more patchy in their distribution than megaripples. Therefore, we might expect that 1) a similar number of sediment grabs in sandwave facies is more likely to include a greater variety of seabed types (and meiofauna) than in megarippled facies and that 2) the more frequent and regular mobility of megaripples tends to favour the presence of fewer meiofauna species and functional groups.

The long-term (i.e. annual to decadal) tendencies for seabed erosion, sediment throughput or sediment accumulation are important because they exert key controls upon habitat type and sediment mobility (Fig. 11). For example, in an environment where sandwaves occur, a long-term tendency towards erosion might increase habitat complexity by producing a series of solitary sandwaves [55] with layers of less mobile sediment between individual bedforms. In contrast, a long-term tendency towards accumulation might be associated with full-bedded sandwaves and lower habitat complexity.

Two main points emerge from our data. Firstly, a broad-scale characterisation of sedimentary environments is unable to account for the microhabitat heterogeneity to which the associated meiofauna will tend to respond. A key aim of future studies should be to incorporate fine-scale spatial information on sedimentary environments into the analysis of broader-scale animal-sediment relationships. For deeper insights, such studies should build on a good conceptual understanding of sedimentary processes operating at a range of spatial and temporal scales, including the movement of sediment across the full range of marine sedimentary bedforms [56], each of which will have important ecological associations (e.g. [81] and our study).

Secondly, small-scale faunal distribution patterns do not always reflect only those marine processes occurring concurrently with sample collection. Whilst this is not a new finding, our results emphasise how important past events can be in affecting animal distributions. Caution must be exercised when attempts are made to explain spatial distributions of animals from physical or biological conditions measured at a single point in time. For example, analyses conducted of pre-storm samples from our facies 8 (‘Sandwaves over longitudinal furrows’) would inevitably generate different faunal results, and hence differing expectations regarding state, than analyses of samples collected shortly after major storms. Further, for those areas where longitudinal furrows occur, there would be relatively high variation in sediment types after storm-associated currents which activate the furrows, but the degree of variation would change through time. Within the furrows themselves, sediments would accumulate relatively rapidly and become finer through time, favouring mud-dwelling meiofauna whereas immediately outside the furrows, the sediments would change in completely different fashion in response to the re-development of ripples and megaripples, with associated different meiofaunal responses.

## Conclusions

The variety of sedimentary regimes at and around Broken Bank and Swarte Bank clearly shapes the meiofaunal nematode communities, and the interaction of local hydrodynamics and sediment transport is clearly a key driver of the processes involved. Their influence occurs in a variety of different ways, and, importantly, in ways that would not have been predictable from using 1) existing generalised theories of seabed disturbance based on frequency or intensity alone [64], [65], [82], as opposed to including duration, period since previous event, etc., 2) sediment summary descriptors alone, as opposed to sedimentary facies, nor using 3) faunal analyses based on taxonomic relationships alone, as opposed to including functional traits.

Our focus on diverse assemblages of organisms with high turnover times (i.e. meiofaunal nematodes), inhabiting highly dynamic sedimentary environments (i.e. subtidal sandbanks and inter-bank swales), has revealed interesting animal-sediment relationships worthy of investigation by future studies of these and a broader range of marine environments. Results from such studies should facilitate a new generation of hypotheses concerning the relative importance of different environmental drivers in structuring benthic communities, and movement towards their rigorous testing. This will provide better indications of the stability of the sediments that benthic communities inhabit and allow better insights into how such communities may respond to natural sediment mobility and to anthropogenic disturbance. Taking account of the past evolution and modern processes of the sedimentary environment is paramount. Contemporary faunal distribution patterns reflect the integrated response of species and functional groups to a wide suite of sedimentary processes, so that effective descriptors of those sedimentary processes will help reveal meaningful animal-sediment relationships.

As noted in the introduction, the EU Habitats Directive requires that “management plans be created in order to detect human-induced change”; such words are easy to write but are likely to be very difficult to achieve. In the case of the UK's candidate Special Areas of Conservation (SAC), which include subtidal mobile sandbanks, there is much to be learnt before such plans can realistically be formed. Our scoping study has indicated some physically and ecologically meaningful measures of seabed physical texture that will help improve seabed characterisation and associations with benthic assemblages.

## Supporting Information

Table S1 **Nematode species abundance by sampling station.**
(XLS)

Table S2 **Relative weight of sediment [%] by grain size [μm].**
(XLS)
